# ^1^H NMR-Based Metabolomics in Pediatric Acute Lymphoblastic Leukemia: A Pilot Study of Plasma and Cerebrospinal Fluid Profiles

**DOI:** 10.3390/metabo16030160

**Published:** 2026-02-28

**Authors:** Agata Serrafi, Małgorzata Pupek, Łukasz Lewandowski, Anna Janicka-Kłos, Andrzej Wasilewski, Adrian Kasprzak, Agnieszka Matera-Witkiewicz, Tomasz Zatoński, Katarzyna Połtyn-Zaradna, Milena Ściskalska, Tomasz Brutkowski, Bernarda Kazanowska

**Affiliations:** 1 Department of Chemistry and Immunochemistry, Wroclaw Medical University, ul. M. Sklodowskiej-Curie 48/50, 50-369 Wroclaw, Poland; 2 Department of Medical Biochemistry, Wroclaw Medical University, ul. T. Chałubińskiego 10, 50-368 Wrocław, Poland; lukasz.lewandowski@umw.edu.p; 3 Department of Basic Chemistry, Wroclaw Medical University, ul. Borowska 211A, 50-556 Wroclaw, Poland; anna.janicka-klos@umw.edu.p; 4 Student Scientific Association of Medical Chemistry and Immunochemistry, Wroclaw Medical University, 50-369 Wroclaw, Poland; adrian.kasprzak@student.umw.edu.pl; 5 Wroclaw Medical University Biobank, Screening of Biological Activity Assays and Collection of Biological Material Laboratory, Wroclaw Medical University, ul. Borowska 211A, 50-556 Wrocław, Poland; agnieszka.matera-witkiewicz@umw.edu.pl; 6 Department and Clinic of Otolaryngology, Head and Neck Surgery, Wrocław Medical University, Borowska 213, 50-556 Wrocław, Poland; tomasz.zatonski@umw.edu.pl; 7 Department of Social Medicine, Wroclaw Medical University, 50-345 Wrocław, Poland; katarzyna.poltyn-zaradna@umw.edu.pl; 8 Department of Pharmaceutical Biochemistry, Wroclaw Medical University, Borowska 211a, 50-556 Wroclaw, Poland; milena.sciskalska@umw.edu.pl; 9 Department of Bone Marrow Transplantation, Pediatric Oncology and Hematology, Wroclaw Medical University, ul. Borowska 213, 50-556 Wrocław, Poland; tomasz.brutkowski@umw.edu.pl (T.B.); bernarda.kazanowska@umw.edu.pl (B.K.)

**Keywords:** acute lymphoblastic leukemia, metabolomics, nuclear magnetic resonance, pilot study

## Abstract

Background/Objectives: This pilot study aimed to evaluate the metabolic profiles in plasma and cerebrospinal fluid (CSF) of 14 patients with acute lymphoblastic leukemia (ALL) and plasma of a control group, using proton magnetic resonance spectroscopy (^1^H NMR). Methods: Multivariate analysis, including orthogonal partial least-squares discriminant analysis (OPLS-DA), was used to analyze the metabolome composition. Results: Significant differences in plasma metabolic profiles were found between the ALL and control groups. We detected elevated levels of formate, citrate, and glycerophosphocholine (GPC), along with decreased concentrations of glutamine and *myo*-inositol. The OPLS-DA model showed stability, with R^2^Y = 69.7% and Q^2^ = 45.15%. Additionally, we observed differences in chemical shifts for leucine, *myo*-inositol, alanine, phenylalanine, and valine between CSF and plasma in patients with ALL. Conclusions: Our findings suggest that metabolomic analysis with ^1^H NMR is a promising tool for identifying potential molecular biomarkers and for deepening our understanding of metabolic reprogramming in pediatric ALL. The observed metabolic differences highlight the potential involvement of the central nervous system in the disease’s pathophysiology.

## 1. Introduction

Hematologic cancers constitute a diverse group of diseases affecting the blood, bone marrow, and lymphatic system; they are also the most common cancers in children. Leukemia, a malignant neoplasm characterized by the clonal proliferation of hematopoietic stem cells in the bone marrow, remains a major focus of ongoing efforts aimed at refining diagnostic approaches, prognostic assessment, and risk stratification of patients. In this context, metabolomic analyses, particularly using ^1^H NMR spectroscopy, are gaining importance. Metabolic changes are an important component of carcinogenesis and represent a potential target for metabolomic approaches, and profiling the metabolome of cancer cells can provide important information about the extent of carcinogenesis. Broad metabolic insights can lead to the identification of novel metabolic biomarkers with significant clinical utility, providing opportunities for earlier disease detection, improved treatment monitoring, and the development of personalized therapeutic strategies [1,2,3,4,5,6].

Leukemias are the most common malignancies in children; in the USA, approximately 3800 children are diagnosed annually with acute lymphoblastic leukemia (ALL) [6]. Among individuals under 19 years of age, ALL is the most prevalent type, accounting for 25% of all childhood cancers. Acute leukemias are defined by the presence of over 20% blast cells in peripheral blood, leading to a more rapid onset of symptoms compared to chronic leukemias. ALL is the most common pediatric leukemia, with B-cell acute lymphoblastic leukemia (B-ALL) being the most widespread form [7]. It is driven by chromosomal aneuploidies, rearrangements, and point mutations. Approximately 30% of affected children present with high hyperdiploidy, which is frequently associated with mutations in the Ras pathway. The prognosis for B-ALL has significantly improved, with cure rates of 75–85% in children, surpassing adult outcomes [8,9,10,11].

Diagnosis of ALL usually begins with peripheral blood evaluation, often revealing anemia, thrombocytopenia, and leukopenia (though leukocytosis is also possible). A crucial step is the morphological evaluation of bone marrow cells. Lymphoblasts, characteristic of ALL, are homogeneous, with a predominantly round and centrally located nucleus, sparse cytoplasm, absence of Auer rods, and possible vacuolation [12,13,14,15]. Flow cytometry, cytogenetic, and molecular studies of bone marrow aspirate are essential for risk stratification. Metabolic panel, serum uric acid dehydrogenase, and liver function tests are crucial for comprehensive patient profiling [16,17,18]. Cerebrospinal fluid (CSF) testing is primarily used to detect the presence of leukemia cells in the central nervous system (CNS). To confirm infiltration and classify CNS involvement (CNS1, CNS2 or CNS3), it is necessary to determine the number of cells, perform a differential analysis, and conduct a cytological examination [19,20].

Metabolomic analysis commonly utilizes ^1^H NMR spectroscopy and mass spectrometry. ^1^D ^1^H NMR spectroscopy is a cornerstone of metabolomics, providing a foundation for understanding metabolic profiles through the identification and quantification of a wide range of metabolites, crucial in numerous biomedical studies [21,22]. Due to its higher reproducibility and ability to identify unknown substances, ^1^ H NMR was chosen as the preferred analytical method in this study. Furthermore, ^1^H NMR analysis is non-invasive, and in vitro results have shown translatability to in vivo clinical applications. It is worth noting that NMR spectroscopy, unlike mass spectrometry, does not require complex sample preparation, making it more suitable for CSF sample analysis, where material is often limited [22,23,24]. Moreover, NMR’s ability to simultaneously identify a wide range of metabolites without prior selection or labeling is a significant advantage in metabolomic screening studies. Both ^1^H NMR and MS are increasingly being investigated as diagnostic methods with potential applications, including in the diagnosis of leukemia [25].

Beyond standard diagnostic and prognostic methods, ^1^H NMR-based metabolomics offers distinct advantages. Its inherent reproducibility using standardized protocols and minimal sample handling allows for consistent and reliable metabolic fingerprinting. It is excellently suited for longitudinal studies, which allow the same individuals to be observed repeatedly over many years. ^1^H NMR is capable of detecting subtle, yet significant, metabolic perturbations associated with disease states. Unlike targeted assays, NMR provides a comprehensive, unbiased view of the metabolome, enabling the discovery of novel biomarkers that might be missed by conventional methods focusing on predefined analytes. The ability to simultaneously quantify multiple metabolites in complex biological matrices, such as blood plasma and CSF, positions ^1^H NMR as a powerful tool to complement and potentially enhance traditional diagnostic approaches, leading to a more holistic understanding of disease pathophysiology.

Our study aimed to investigate and compare spectral metabolic patterns between children with acute lymphoblastic leukemia and healthy controls using qualitative and semi-quantitative ^1^H NMR spectroscopy. By analyzing metabolic signatures of blood plasma and cerebrospinal fluid samples, we aimed to identify potential biomarker candidates that could help develop future strategies for early diagnosis of acute lymphoblastic leukemia and disease monitoring. Leukemia, a heterogeneous disease characterized by metabolic derangements, is an ideal candidate for metabolomic studies. Our preliminary findings suggest significant differences in metabolic profiles between the two groups. This study represents one of the first applications of ^1^H NMR-based metabolomics in childhood leukemia, offering a novel approach to understanding and treating this complex disease.

Further studies in this area are being conducted with a larger and more homogeneous group of children being treated for ALL to strengthen the initial findings and obtain greater statistical power.

## 2. Materials and Methods

### 2.1. Study Population

This preliminary study included fourteen pediatric patients diagnosed with acute lymphoblastic leukemia, according to the International Classification of Diseases, 10th Revision (ICD-10). Patients were treated per their respective protocols: the AIEOP-BFM-2017 protocol for acute lymphoblastic leukemia (ALL; EudraCT No. 2020-005017-41). All patients received care at the Department of Bone Marrow Transplantation, Oncology, and Hematology, Wroclaw Medical University, between 19 October 2021 and 24 November 2022. Written informed consent was obtained from each patient’s legal guardian after detailed explanation of the cerebrospinal fluid collection procedure and its potential risks and complications. The study adhered to the Declaration of Helsinki and was approved by the Bioethics Committee of Wroclaw Medical University (Approval No. KB-525/2021, approved on 9 June 2021).

Acute lymphoblastic leukemia was diagnosed and classified by analyzing leukemic cell markers using flow cytometry according to standard clinical criteria, identifying 11 patients with B-cell precursor ALL (5 standard risk, 3 moderate risk, 3 high risk) and 2 with T-ALL (high risk). The AIEOP-BFM 2017 protocol was used in the treatment of ALL. Immunophenotyping included a comprehensive panel of antigens (notably CD19, CD10, and cCD3) to ensure precise lineage assignment and subtype identification for risk stratification.

Inclusion criteria were: age ≤ 18 years; a confirmed diagnosis of acute lymphoblastic leukemia; and either first admission or readmission to the hospital for planned oncological treatment (e.g., administration of chemotherapy and/or radiotherapy). Exclusion criteria included a history of medical conditions such as malignancies other than acute lymphoblastic leukemia.

The ALL cohort (*n* = 14; age range 2–16 years, mean 5.7 ± 5.0 years) comprised 8 boys (57%) and 6 girls (43%). These patients were admitted for diagnosis or planned oncological treatment, including chemotherapy and/or radiotherapy. Basic demographic data (age, gender, weight, height), previous medical history, and clinical signs and symptoms were collected for all patients. Clinicopathological parameters included histological confirmation of disease, gender, and age. Selected laboratory values, such as complete blood count with differential (neutrophils, lymphocytes, monocytes), were obtained from plasma samples (see Table 1).

For the control group, twenty healthy children (free of leukemia) aged 11–17 years (mean 14.3 ± 2.0 years), including 8 females and 12 males, were enrolled. These participants were drawn from the “Population Cohort Study of Wroclaw Citizens” (PICTURE), conducted between 2019 and 2021 by Wroclaw Medical University in collaboration with the Wroclaw Municipality [26]. The PICTURE study was approved by the Bioethics Committee of Wroclaw Medical University (Approval No. KB-667/2019).

To confirm the quantitative reliability of ^1^H NMR profiling, levels of commonly used clinical markers of lactate, glucose, and creatinine were compared with data obtained from routine biochemical analyses performed in the laboratory. A significant positive correlation was observed for all three metabolites (Spearman’s coefficient ρ > 0.60, *p* < 0.001 for each), confirming that ^1^H NMR spectroscopy reflects the metabolic status of pediatric patients.

### 2.2. Sampling

Paired CSF and plasma samples were collected from pediatric patients during routine diagnostic lumbar puncture performed for suspected or previously diagnosed acute lymphoblastic leukemia. To avoid the confounding effects of medical intervention, samples were collected from children not undergoing chemotherapy at the time of sampling, either prior to or between cytoreductive therapy sessions.

Lumbar puncture with CSF collection was performed only when clinically indicated to assess central nervous system involvement and guide diagnosis and treatment in selected ALL subtypes. Standard diagnostic lumbar punctures were performed under regional anesthesia. Cerebrospinal fluid was collected aseptically in sterile, screw-capped tubes and immediately used for clinical diagnostics. For this study, CSF samples were then centrifuged at 1500× *g* for 15 min at 4 °C, aliquoted (0.1 mL each), and stored at −76 °C until analysis. Venous blood (2 mL) was drawn from an antecubital vein into calcium-balanced, lithium-heparinized tubes (SARSTEDT Blood Gas Monovette^®^, SARSTEDT Ltd., Leicester, UK). Plasma was obtained by centrifugation at 2000× *g* for 15 min, aliquoted (0.1 mL), and stored at −76 °C [27].

Plasma samples, but not CSF, were collected from healthy controls. Venous blood was collected into K_3_EDTA tubes and centrifuged at 2200× *g* for 15 min, and the resulting plasma was aliquoted (0.4 mL) and stored at −76 °C until analysis. For this study, CSF samples were then centrifuged in 1500-balanced, lithium-heparinized tubes (SARSTEDT Blood Gas Monovette^®^, SARSTEDT Ltd., Leicester, UK). Plasma was obtained by centrifugation in 2000-EDTA tubes, centrifuged at 2200× *g*.

Prior to analysis, frozen samples were thawed for 1 h at 20 °C to ensure complete dissolution. Freeze–thaw cycles were strictly avoided due to their detrimental impact on sample integrity. Hemolyzed, icteric, or lipemic specimens were discarded.

### 2.3. Acquisition of NMR Spectra

Blood plasma was thawed on ice and subsequently centrifuged. A 50 μL aliquot of the clear plasma supernatant was collected and mixed with 10 μL of deuterated water (D_2_O). The resulting 60 μL mixture was then transferred at room temperature into a 5 mm NMR tube for spectroscopic analysis. All Nuclear Magnetic Resonance (NMR) experiments were performed on a Bruker AVANCE III™ 600 MHz spectrometer, equipped with a 1.7 mm TCI cryoprobe ([^1^H, ^13^C, ^15^N]).

Several experimental techniques were employed to mitigate the challenge of broad resonances originating from proteins and protein–lipid complexes in plasma, which can obscure relevant metabolite signals. Specifically, diffusion-edited NMR and T2 filtering sequences were implemented to suppress macromolecular signals and enhance the detection of small-molecule metabolites. In forthcoming studies, we plan to further explore protein precipitation as an alternative pre-analysis approach to improve spectral quality and interpretability.

Spectra were acquired at 298 K. One-dimensional proton spectra were collected using both zgesgp and noesygppr1d (^1^D NOESY) pulse sequences. Water signals in the spectra were suppressed using either an excitation sculpting pulse sequence or presaturation. For both sequences, an exponential window function with a line broadening factor of 0.2 Hz was applied to the free induction decay (FID) before Fourier transformation. For each FID, 32 scans were collected with a spectral width of 12 ppm, an acquisition time of 4.5 s, a relaxation delay of 5 s, and a mixing time of 10 ms. All NMR spectra were phased and baseline-corrected using TopSpin software (version 3.6.5, Bruker BioSpin, Rheinstetten, Germany). Parameters were optimized to ensure accurate quantification of metabolites in subsequent analyses using Bruker TopSpin (Bruker TopSpin 5.0) and MestReNova software.

### 2.4. NMR Profiling and Metabolite Determination

Identification and qualitative assessment of the detected metabolites were performed using MestReNova (version 8.1.4-12489) for profiling and confirmed with TopSpin software. MestReNova facilitates the deconvolution of metabolites in complex samples and enables concentration determination even in spectral regions with overlapping peaks. The software matches detected peaks to a set of model spectra, characterizing the chemical environments of each metabolite. An identical volume of D_2_O was added to all blood plasma and CSF samples to provide a deuterium lock signal for NMR spectroscopy. Proton peak assignments for individual amino acid residues and other substances were made for all acquired spectra. Further information on proton peak assignments was obtained by comparing chemical shifts with those available in the Human Metabolome Database (HMDB: http://www.hmdb.ca, accessed on 24 August 2023).

### 2.5. Statistical Analysis

Preprocessing and statistical analysis were performed using Python 3.10.7, leveraging packages such as NumPy, Pandas, and SciPy. Given the relatively small sample size, non-normal distribution (confirmed by the Shapiro–Wilk test), and the presence of outliers (identified via Q-Q plots), a non-parametric statistical approach was adopted. Differences in analyzed variables between the control and ALL groups were assessed using the Mann–Whitney U test. To evaluate differences in metabolite chemical shifts between plasma and cerebrospinal fluid in ALL patients, the Wilcoxon test was employed, with pairwise ties handled according to J.W. Pratt [28] and normal approximation as described by E.E. Cureton [29,30,31,32,33,34,35,36,37,38,39,40]. Qualitative variables (e.g., gender, disease status) were presented as percentages, while continuous variables were reported as medians (1st and 3rd quartiles). Statistical significance was defined as a *p*-value less than 0.05.

For multivariate analysis, data were subjected to principal component analysis (PCA). To further enhance group separation and identify discriminatory features, partial least-squares discriminant analysis (PLS-DA) and orthogonal partial least-squares discriminant analysis (OPLS-DA) were additionally performed.

### 2.6. Precision and Sample Size in the Pilot Investigation

In this pilot study comprising 14 cases and 20 controls, the following precision estimates were obtained: (1) a 95% confidence interval (CI) half-width of 0.68 for the standardized mean difference (d); (2) conservative 95% margins of error of 26% (cases; 25% with finite population correction) and 22% (controls) for binomial parameters (e.g., sensitivity and specificity); and (3) AUC 95% CI half-widths of approximately 0.12–0.19 for plausible AUC values. The modest group-size imbalance incurs an information loss of about 3% relative to a balanced design of the same size. These estimates are consistent with the aims of a pilot study, namely, to assess variability and inform the sample-size determination of the subsequent definitive trial. While the sample size in this study is relatively small (for the ALL group), it is consistent with the recommendations for pilot studies, which typically require between 10 and 35 participants to evaluate feasibility and provide preliminary data for power calculations in subsequent research.

## 3. Results

Descriptive plasma laboratory parameters (expressed as median values) for the ALL and healthy control groups are shown in Table 1. Compared with controls, patients with ALL showed significantly lower white blood cell count (WBC; *p* = 0.003) including neutrophils (*p* < 0.001), red blood cell count (RBC; *p* < 0.001), hemoglobin concentration (HGB; *p* < 0.001), hematocrit (HCT; *p* < 0.001) and platelet count (PLT; *p* = 0.006). Conversely, the proportions of lymphocytes were significantly higher in ALL patients (*p* = 0.02). White blood cell counts may be very high, exceeding 100,000 per mm^3^ in some subtypes of leukemia. However, they may also be low (leukopenia), as observed in our study group, particularly in the early phase of the disease when the bone marrow is heavily infiltrated. We also observed decreased red blood cell counts (anemia) and platelet counts (thrombocytopenia), accompanied by abnormal MCHC and MPV values as a consequence of disruption of hematopoiesis in ALL.

**Figure 1:**
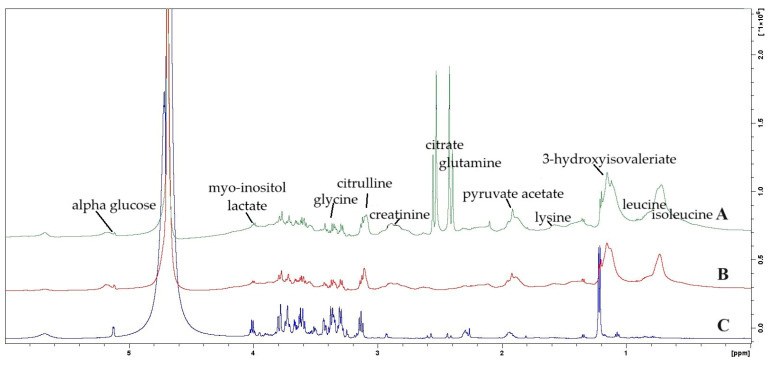
Comparison of representative 600 MHz ^1^H NMR spectra and metabolite profiles of study samples: (A) plasma from a healthy control; (B) plasma from a patient with ALL; (C) cerebrospinal fluid from a patient with ALL.

Examples of typical 600 MHz ^1^H NMR spectra are presented in Figure 1, and representative metabolite assays from CSF and plasma of children with leukemia, along with control plasma samples, are detailed in Table 2 and Table 3.

Chemical shift analysis, using NMR profiling and metabolite determination, allowed the identification of 26 compounds in the analyzed blood plasma samples. Of these, arginine, α-H1-glucose, β-H2-glucose, creatinine, alanine, lactate, valine, leucine, isoleucine, glycine, histamine, uridine diphosphate-glucose, guanosine monophosphate, phosphocholine, and citrulline showed no statistically significant differences (Mann–Whitney U test) in chemical shifts between the leukemic plasma and healthy control groups. However, other metabolites showed small but statistically significant differences in chemical shift values. Specifically, the chemical shift values of acetone, β-hydroxybutyrate, glutamine, phenylalanine, lysine, *myo*-inositol, and *scyllo*-inositol were significantly lower in leukemic plasma compared to healthy control plasma. Conversely, the chemical shift values of formate, citrate, acetoacetate, and glycerophosphocholine (GPC) were significantly higher in leukemic plasma (each difference with *p* < 0.001, Table 2).

Chemical shift analysis (Figure 1 and Table 4), performed through NMR profiling and metabolite determination, allowed for the identification of 16 compounds common to both CSF and plasma samples from children with leukemia. Among these, acetone, arginine, citrate, citrulline, creatinine, formate, glutamine, glycine, lactate, lysine, and *scyllo*-inositol were identified in both CSF and plasma with identical chemical shift values. Other metabolites, however, exhibited small but statistically significant differences in their chemical shift values. Specifically, the chemical shift values in CSF for leucine and *myo*-inositol were significantly lower, while those for alanine, phenylalanine, and valine were significantly higher, compared to their respective chemical shift values in plasma (*p* < 0.001 for each difference). These findings suggest alterations in the microenvironment or molecular interactions affecting these metabolites in the blood plasma of ALL patients (Table 3).

**Figure 4:**
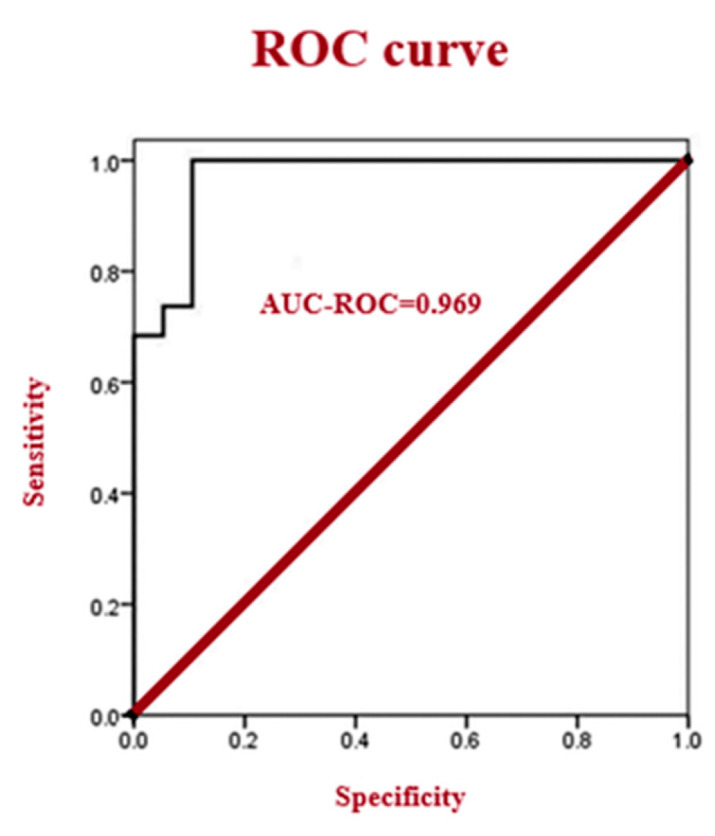
ROC curve assessment of choline and tyrosine as combined biomarkers in the diagnosis of ALL.

Metabolite identifications were based on their characteristic chemical shift values, expressed in parts per million (ppm), obtained from 600 MHz ^1^H NMR spectra (exemplified in Figure 4) and compared against the Human Metabolome Database (HMDB) reference values (Table 4). For instance, alanine was identified by a chemical shift around 1.455 ppm in both plasma and CSF, while glucose was identified by its α-H1 peak at approximately 5.190 ppm and β-H2 peak at 3.280 ppm. The observed slight but significant differences in chemical shift values for certain metabolites underscore changes in their electronic environment in leukemia plasma.

A total of 26 compounds were identified in blood plasma, 16 of which were common to both cerebrospinal fluid and plasma. Table 5 provides a complete list of compounds, with particular emphasis on compounds specific to individual biological matrices, and provides links to the Human Metabolome Database (HMDB).

**Figure 2:**
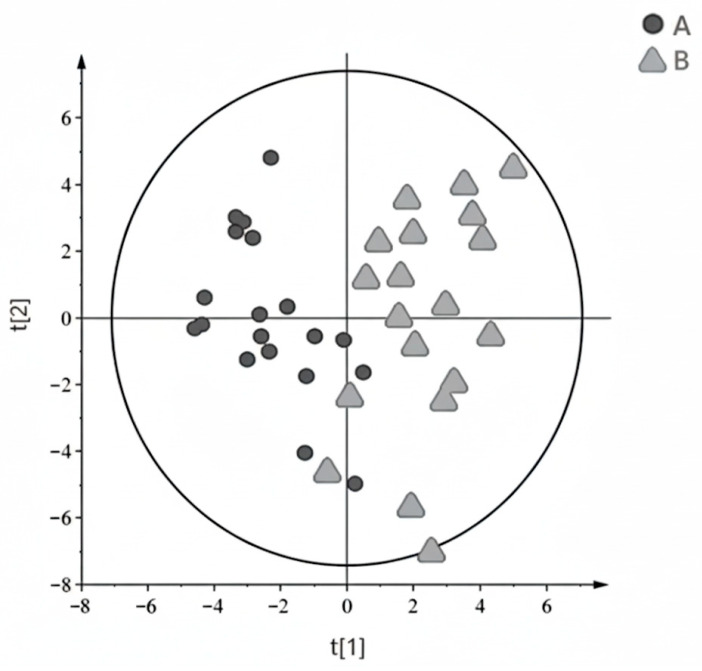
The OPLS-DA score plot shows the results of multivariate analysis performed on plasma data from controls (circles, A) and ALL patients (triangles, B).

Multivariate analysis is a powerful tool commonly employed to identify subtle differences between groups within complex datasets. In this study, unsupervised principal component analysis (PCA) was initially utilized for exploratory data analysis. However, the first two principal components of the PCA model did not clearly distinguish between healthy controls and ALL patients. To achieve better discrimination, we subsequently employed supervised orthogonal partial least-squares discriminant analysis (OPLS-DA). The OPLS-DA model showed a moderate separation between the groups (Q2 = 0.45). To ensure the validity of the model and mitigate the risk of overfitting, common in small cohorts (*n* = 14 vs. *n* = 20), cross-validation was performed, confirming the model’s preliminary predictive power. The results of this analysis are visually represented in Figure 2. But the ALL group was considerably younger (median 3.5 years) compared to the control group (median 15 years) (*p* < 0.001) (Table 1 and Table 6). Furthermore, data scatter is likely exacerbated by different clinical forms of the disease (11 B-ALL cases vs. 2 T-ALL cases).

The results (Table 6) show that signal intensity of certain metabolites, such as formate and glucose, exhibits a correlation with age within the studied age range (R^2^ = 0.73 and 0.65, respectively). In contrast, other metabolites—including arginine, valine, lactate, and glutamine—showed low R^2^ values (0.02 to 0.20), suggesting that their signals are largely independent of age in this cohort. By identifying these age-independent metabolites, we can more reliably propose them as potential biomarkers of pediatric ALL, as their variability is less likely to be influenced by natural development. This statistical step was essential to ensure that the reported differences between ALL and controls are not merely reflections of chronological age. On the other hand, other metabolites, including arginine, valine, lactate, and glutamine, do not show a strong association with age, as indicated by low R^2^ values (ranging from 0.02 to 0.20). This indicates that their chemical shifts are largely independent of age, making them potentially better biomarkers of the disease, as their variability is less related to natural maturation processes. Using linear regression analysis allows for data correction by removing the effect of age from metabolite chemical shifts. This allows for more precise and reliable results regarding metabolic differences between groups (e.g., ALL vs. controls) and the selection of biomarkers that are more specific to the disease entity itself than to age-related changes.

**Figure 3:**
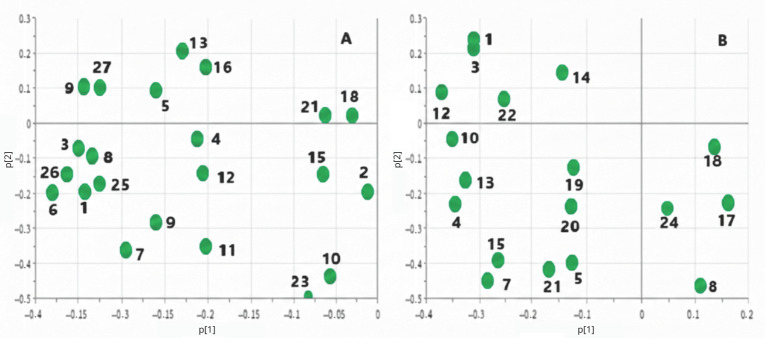
PLS-DA loading plots of (**A**) blood plasma and (**B**) cerebrospinal fluid of leukemia patients. (1) Alanine; (2) arginine; (3) histidine; (4) glutamine; (5) lysine; (6) tyrosine; (7) valine; (8) phenylalanine; (9) citrulline; (10) creatine; (11) acetate; (12) acetone; (13) lactate; (14) 3-hydroxyisovalerate; (15) 2-hydroxybutyrate; (16) glucose; (17) myo-inositol; (18) uridine diphosphate-glucose; (19) glycerophosphocholine; (20) guanosine monophosphate; (21) phosphocholine; (22) GABA; (23) glycine; (24) *Scyllo*-inositol; (25) formate; (26) citrate; (27) isobutyrate.

Analysis of PLS-DA loading plots (Figure 3) revealed several key plasma metabolites that differentiate ALL samples from other groups, including control samples (Figure 2A). In cerebrospinal fluid, lysine was identified as a significant marker distinguishing ALL patients from other groups (Figure 2B). Interestingly, none of the identified metabolites effectively discriminated healthy controls from leukemia patients (Figure 3).

Logistic regression, a probabilistic non-linear regression analysis, was employed. Given its high predictive power, it is widely utilized in epidemiological studies. Stepwise logistic regression with backward selection was implemented to identify potential diagnostic biomarkers among the differential metabolites. The logistic regression model was validated by assessing its parameters and evaluating the diagnostic performance of the resulting equations (Table 1, Table 2 and Table 3). Choline and tyrosine were identified as the optimal combination for diagnosing ALL patients. Based on data from ALL and control subjects, ROC curve analysis incorporating choline, tyrosine, and unsaturated lipids yielded an area under the curve (AUC) of 0.969 for the overall conclusive determination between the two groups (Figure 4). An AUC closer to 1 reflects superior diagnostic performance. The diagnostic model based on choline and tyrosine demonstrated high accuracy in distinguishing ALL patients from the control group.

A comparison of metabolite chemical shifts in the cerebrospinal fluid and blood plasma of children with ALL was performed using the Wilcoxon signed-rank test (Table 3). While acetone (*p* = 0.060), arginine (*p* = 0.902), citrate (*p* = 0.031), citrulline (*p* = 0.382), creatinine (*p* = 0.177), formate (*p* = 1.000), glutamine (*p* = 0.822), glycine (*p* = 0.084), lysine (*p* = 0.708), and *scyllo*-inositol (*p* = 0.157) showed similar values in both fluids, alanine, phenylalanine, and valine exhibited significantly higher values in CSF (*p* < 0.001 for each). Conversely, lactate (*p* = 0.002), leucine (*p* < 0.001), and *myo*-inositol (*p* < 0.001) demonstrated significantly lower chemical shifts in CSF. These findings may indicate distinct metabolic processes occurring in the central nervous system compared to blood in these patients. CSF metabolites were identified by matching their characteristic chemical shifts in the ^1^H NMR spectra (Figure 4) with reference values from the HMDB (Table 4), exemplified by lactate around 4.107 ppm and *myo*-inositol around 4.070 ppm. Initial principal component analysis (PCA) did not clearly distinguish between the groups. However, subsequent orthogonal partial least-squares discriminant analysis (OPLS-DA) (Figure 3) successfully separated the groups (R2X = 51.3%, R2Y = 69.7%, Q2 = 45.15%). Importantly, backward selection logistic regression analysis identified choline and tyrosine as optimal potential diagnostic biomarkers. The model, which also included unsaturated lipids, achieved a high area under the receiver operating characteristic curve (AUC = 0.969), indicating strong performance in distinguishing patients with ALL from healthy children.

## 4. Discussion

In this pilot study, ^1^H NMR spectroscopy was used to analyze the metabolomic profiles of cerebrospinal fluid and blood plasma in children with acute lymphoblastic leukemia, and blood plasma in healthy controls. Qualitative analysis of the ^1^H NMR spectra led to the identification of over 33 metabolites, with 26 distinct compounds detected in blood plasma. Notably, the spectra from ALL patients significantly differed from those from healthy children, exhibiting subtle but statistically significant variations in chemical shift values for specific metabolites. Furthermore, the metabolite composition of cerebrospinal fluid (CSF) was distinct from that of plasma, revealing both shared and unique compounds (Table 4, Figure 1 and Figure 2). This research is among the first to utilize ^1^H NMR-based metabolomics to analyze such pediatric ALL profiles, offering valuable insights into the disease’s metabolic landscape [22,23,24,25,26].

A systematic approach was employed to identify metabolites, utilizing the Human Metabolome Database (HMDB) and the existing literature to ensure the accurate assignment of unassigned peaks. To provide a comprehensive clinical view, it is essential to highlight the distribution of these substances across different biological matrices (Table 5).

Despite these observations, the initial PCA showed a lack of clear separation, which may be attributed to age disparities between the younger ALL patients (median 3.5 years) and older controls (median 15 years) (Table 6). Additionally, clinical heterogeneity—predominantly B-ALL (11 cases) versus T-ALL (2 cases)—may contribute to data scatter. For improved transparency, lymphoid cell counts for all 14 cases are included in the Supplementary Material.

The metabolic shifts revealed in this study may be primarily explained by the heavily altered metabolism of cancer cells (Table 7). While interpreting individual metabolites is informative, a complex pattern specific to ALL emerges involving a “*triad*” of choline, tyrosine, and unsaturated lipids. Biochemically, this specific combination reflects the synergistic requirements of leukemic lymphoblasts. The elevation of choline-containing compounds like glycerophosphocholine (GPC) may reflect accelerated membrane turnover and phospholipid remodeling—a hallmark of rapidly dividing leukemic cells. Simultaneously, the depletion of tyrosine underscores its intensive consumption as a precursor for protein synthesis and signaling molecules, while fluctuations in unsaturated lipids suggest a shift toward membrane structural integrity and signaling lipid synthesis to support tumor survival under metabolic stress. This complex pattern may serve as a specific metabolic “*signature*” that captures the multifaceted nature of leukemic reprogramming more effectively than single markers [22,31,32].

A critical point for discussion is the advantage of ^1^H-NMR over other techniques like routine HPLC or mass spectrometry (MS). While MS offers higher sensitivity, ^1^H-NMR provides an “*unbiased*” and comprehensive view of the metabolic landscape, allowing for the simultaneous detection of amino acids, organic acids, and sugars in a single experiment. Unlike HPLC, which often requires separate chromatography runs for different chemical classes (e.g., one for amino acids and another for lipids), NMR’s high reproducibility and non-invasive nature make it superior for identifying complex patterns in limited clinical samples like CSF. This ability to capture a broad metabolic fingerprint without prior selection of analytes is what enabled the discovery of the choline–tyrosine–lipid triad as a potential diagnostic tool [41,42]. The findings revealed significant alterations within the glucose–alanine pathway. Elevated chemical shifts of formate and citrate indicate associations with the Warburg effect and disruptions in the tricarboxylic acid (TCA) cycle.

Leukemic cells exhibit unique metabolic traits, including increased glucose uptake (exploited in FDG-PET scans) and upregulated mTOR and PKM2 pathways [22,31,32,43,44,45,46,47,48,49,50]. Beyond glucose, amino acids like glutamine fuel the TCA cycle under limiting conditions, while branched-chain amino acids (BCAAs) like leucine and valine are increasingly recognized for their role in nutrient acquisition [41,51,52,53,54,55].

Barriers protecting the central nervous system, such as the blood–brain barrier (BBB), ensure homeostasis. The distinct metabolic profiles between CSF and plasma (e.g., higher phenylalanine and valine in CSF) are not merely reflections of systemic changes but direct indicators of the unique metabolic microenvironment within the CNS, potentially caused by leukemic cell infiltration or barrier dysfunction [41,42,56,57].

Several limitations must be acknowledged. The analysis of plasma was challenged by broad signals from macromolecules; while diffusion-edited NMR and T2 filtering were used to mitigate this, further optimization, like protein precipitation, is needed. Additionally, this pilot study featured a small cohort to facilitate the initial identification of biomarkers. The invasive nature of CSF collection remains an ethical concern, though samples were obtained only when clinically indicated.

In conclusion, this pilot study demonstrates that ^1^H NMR-based metabolomics holds promise as a complementary tool for the diagnosis and monitoring of pediatric ALL. Future research should integrate these findings with transcriptomic and proteomic data to move toward personalized medicine, validating these biomarkers in larger, more homogeneous cohorts.

### 4.1. Potential Metabolic Biomarkers: A Deeper Analysis

Based on the pilot study, the most promising metabolites, showing the largest and statistically significant differences in resonance intensities and calculated concentrations (*p* < 0.001), are those identified in the blood plasma of ALL patients. While the OPLS-DA models provided moderate predictive value (Q^2^ ≈ 0.45), the significance of these findings is confirmed by univariate analysis. In ALL patients, significantly lower signal intensities of acetone, β-hydroxybutyrate, glutamine, phenylalanine, lysine, *myo*-inositol, and *scyllo*-inositol were found. The decrease in ketone bodies and glutamine likely reflects the intensified metabolic demand and altered energy substrate utilization by leukemic cells. Reduced signal intensities of *myo*-inositol and *scyllo*-inositol may further indicate changes in cell signaling or membrane integrity specific to the leukemic environment. While amino acids are often intensively consumed by rapidly proliferating cancer cells for the synthesis of proteins, nucleotides, and other cellular components. *Myo*-inositol and *scyllo*-inositol play a role in cell signaling and membrane integrity, and their reduced levels may express changes in cell membrane structure or signaling pathways in cancer cells. At the same time, significantly high peaks of formate, citrate, acetoacetate, and glycerophosphocholine were observed. An elevated signal intensity of formate may be associated with increased activity of one-carbon pathways, which are crucial for nucleotide synthesis and other biosynthetic processes necessary for rapid cancer cell growth.

Elevated signal intensities of citrate, a key intermediate of the Krebs cycle [41], in plasma may reflect changes in the regulation of the metabolic cycle or its allocation for lipid biosynthesis, which is typical for cancer cells. An increase in the acetoacetate peak may also indicate deregulation of energy metabolism, although interpretation requires further research in the context of specific metabolic pathways in leukemia. Elevated peaks of choline-containing compounds, such as GPC, are often observed in cancers and are associated with increased cell membrane turnover and cell proliferation. Additionally, in a comparison of cerebrospinal fluid [41,56] and plasma in patients with hematological malignancies, significant differences were observed, suggesting a distinct metabolic environment in the central nervous system. The lower CSF group exhibited significantly lower peaks for leucine and *myo*-inositol, alongside elevated levels of alanine, phenylalanine, and valine. Comparison of cerebrospinal fluid and plasma revealed a distinct metabolic environment within the CNS. These CSF-specific alterations may serve as potential biomarkers for monitoring CNS involvement. Metabolites showing high significance (*p* < 0.001) warrant further validation in larger, homogeneous cohorts to assess their utility as diagnostic or prognostic biomarkers in pediatric hematological malignancies.

Additionally, a comparison of the cerebrospinal fluid and plasma metabolomes in ALL patients reveals critical insights into the compartmentalization of the disease. From a clinical perspective, certain substances were identified as unique to each biofluid, reflecting the distinct metabolic demands of the systemic circulation and the central nervous system. For instance, glycerophosphocholine and histamine were predominantly detected in plasma, suggesting their role in systemic inflammatory responses and membrane turnover. Conversely, the relative presentation of branched-chain amino acids (BCAAs) and phenylalanine showed significant variation; higher relative concentrations in CSF compared to plasma may indicate a disruption of the blood–brain barrier (BBB) or direct metabolic activity of leukemic cells infiltrating the CNS. Identifying these unique signatures is essential for clinical monitoring, as the presence of plasma-typical metabolites in the CSF could serve as an early indicator of CNS involvement, potentially guiding more aggressive intrathecal therapy.

### 4.2. Potential Practical Applications of Metabolic Biomarkers in Childhood Leukemia Treatment

Beyond the general statement about clinical potential, the identified metabolic biomarkers, though currently preliminary and based on models with moderate predictive power at this pilot stage, hold promising prospects for practical application in the management of pediatric leukemia. Integrating metabolomics into routine clinical practice could significantly enhance patient care in several key areas.

Metabolomics offers the possibility of detecting subtle biochemical changes that precede clinically overt symptoms of leukemia relapse. Regular monitoring of key metabolite concentrations in plasma, such as decreased glutamine or increased formate, could serve as a highly sensitive and specific indicator of an impending relapse. Such an approach would enable earlier detection of minimal residual disease (MRD) at the metabolic level, allowing for more timely implementation of therapeutic interventions before the disease reaches an advanced stage, thereby potentially improving prognosis [57].

Such an approach would enable highly sensitive early detection of minimal residual disease (MRD) at the metabolic level, facilitating the timely implementation of therapeutic interventions before disease progression, potentially improving patient prognosis.

Dynamic changes in a patient’s metabolic profile during chemotherapy or other treatments could provide objective measures of treatment effectiveness. For example, the normalization of acetone or *myo*-inositol levels after the initiation of therapy could indicate a positive response to treatment. Real-time monitoring of these changes could enable clinicians to more quickly optimize drug dosages, modify treatment protocols, or make earlier decisions to change therapy in cases of non-response, thereby minimizing unnecessary burden on the patient’s body [56,57].

The identified differences in metabolites can help in identifying subgroups of patients with varying risks of disease progression, specific complications, or responses to particular drugs. For instance, a unique metabolic profile, characterized by specific levels of phenylalanine or glycerophosphocholine, might indicate patients requiring more intensive therapy, or those who are more susceptible to adverse effects. Such an approach, based on a personalized metabolic profile, could pave the way for more precise and targeted therapy, maximizing its effectiveness while minimizing toxicity.

Leukemia may already involve the central nervous system at the time of diagnosis, which requires a specific therapeutic approach. Differences in metabolites between cerebrospinal fluid and blood plasma (e.g., for leucine or alanine) could potentially serve as less invasive indicators of CNS involvement in leukemia. The development of biomarkers in CSF could reduce the need for frequent, invasive lumbar punctures, while simultaneously improving the ability to monitor and effectively treat CNS disease [56,57].

It should be noted that due to the relatively small sample size, these OPLS-DA models carry a risk of overfitting. Therefore, the reported Q2 values should be interpreted as moderate, and these preliminary results must be validated using larger, independent cohorts to confirm their diagnostic robustness.

In this pilot study, qualitative and preliminary quantitative ^1^H NMR spectroscopy was applied to characterize plasma and cerebrospinal metabolic composition. While chemical shifts (ppm) were used to confirm molecular identity and environmental changes, the reported differences in metabolite levels refer to their quantitative abundance derived from signal integrals.

### 4.3. Limitations

A primary limitation of this study is the relatively small and single-center cohort, which may affect the generalizability of the findings. Most importantly, we acknowledge a significant age disparity between the ALL group (mean 5.4 ± 4.3 years) and the healthy control group (mean 14.3 ± 2.0 years). This difference represents a substantial confounding factor in pediatric metabolomics, as metabolic profiles are known to evolve during physiological maturation. While we performed exploratory linear regression analysis on simulated data to assess age–metabolite correlations, we recognize that such mathematical adjustments do not fully compensate for the actual biological bias inherent in non-comparable age groups. As a pilot investigation, these results serve as a proof-of-concept for the utility of ^1^H NMR-based metabolomics in pediatric ALL.

## 5. Conclusions

Recent cancer research underscores the critical role of altered metabolism, especially glucose metabolism, in tumor development. A comprehensive understanding of these metabolic shifts is vital for devising novel therapeutic strategies. This pilot study employs qualitative ^1^H NMR spectroscopy to profile the metabolic landscape and identify representative spectral patterns across various acute leukemia subtypes. The preliminary findings indicate that ^1^H NMR-based metabolomics can distinguish overall metabolic fingerprints of children with leukemia from those of healthy controls. As a pilot investigation, these results prioritize the exploration of spectral variations over absolute metabolite quantification. The preliminary findings from this study indicate that ^1^H NMR-based metabolomics can indeed distinguish the metabolic profiles of children with leukemia from those of healthy controls. However, challenges persist, particularly in plasma sample analysis and due to patient group heterogeneity.

We acknowledge some limitations inherent in this pilot study. Analysis of plasma samples is challenging due to macromolecular interference. Furthermore, to increase statistical power and facilitate detailed comparisons, future studies should focus exclusively on a single, precisely defined subtype of acute lymphoblastic leukemia. We also acknowledge the invasive nature of cerebrospinal fluid collection, which was performed only when clinically indicated.

Despite these obstacles, our findings highlight the potential of this methodology to discover new biomarkers and advance our understanding of the complexities of metabolic hematologic malignancies in children. To strengthen these preliminary results and increase the impact and reliability of the study, future work should include robust validation. This validation will consist of: refining the methodology by improving plasma sample preparation techniques and optimizing NMR spectral acquisition to enhance metabolite detection and quantification; increasing sample size, meaning conducting studies with larger, more homogeneous patient cohorts, carefully stratified by ALL subtype and treatment phase to enable detailed, statistically reliable comparisons; and utilizing advanced techniques such as targeted metabolomics or high-performance liquid chromatography to provide absolute quantification and confirm our initial qualitative findings. Such coordinated efforts, conducted with improved methodologies and larger, more homogeneous patient cohorts, are crucial to fully realizing the clinical potential of metabolomics in pediatric hematologic malignancies, ultimately leading to improved diagnosis, prognosis, and therapeutic stratification.

The identification of these potential biomarkers is further supported by the high consistency observed between our NMR data and standard clinical laboratory markers. By comparing common metabolites such as lactate, glucose, and creatinine, we demonstrated that ^1^H NMR results align with established clinical diagnostics. This agreement not only validates our spectroscopic methodology but also suggests that metabolomics can reliably complement routine clinical assessments in pediatric leukemia.

In conclusion, our pilot study identifies significant metabolic alterations in children with ALL. Future large-scale, multi-center, and longitudinal studies are required to validate these metabolites as robust clinical biomarkers and to correlate them with long-term clinical outcomes such as relapse-free survival and therapeutic toxicity.

## Figures and Tables

**Table 1:** Comparison of the median (1st–3rd quartile) plasma laboratory parameters between children with ALL and healthy controls.

Parameter, Unit	ALL (*n* = 14)		Controls (*n* = 20)		*p* Level	Reference Range
	*n*	Median (1st, 3rd Quartiles)	*n*	Median (1st, 3rd Quartiles)		
Age, years	14	3.5 (2.5, 6.6)	20	15.0 (12.5, 16.0)	<0.001	n/a
WBC, ×10^9^/L	10	1.8 (0.9, 3.6)	20	5.2 (4.4, 6.0)	0.003	* 5–10
Neutrophils, %	10	21.0 (6.0, 41.4)	20	44.7 (39.2, 51.3)	<0.001	50–70
Lymphocytes, %	10	59.0 (41.8, 83.6)	20	40.9 (33.5, 46.8)	0.02	20–45
Monocytes, %	10	7.9 (3.4, 33.2)	20	9.9 (8.6, 11.5)	0.681	0.0–10.0
HCT, vol%	10	25.6 (23.7, 27.4)	20	41.0 (39, 44)	<0.001	* 32–44
RBC, ×10^12^/L	10	3.0 (2.8, 3.2)	20	4.8 (4.7, 4.9)	<0.001	* 4.0–5.5
HGB, g/dL	10	8.7 (8.5, 9.6)	20	13.7 (13.1, 14.7)	<0.001	* 9.5–15.5
MCV, ×10^15^/L	10	83.9 (82.8, 87.5)	20	86.6 (81.9, 88.9)	0.779	80–97
MCH, ×10^12^/g	10	29.6 (28.8, 30.0)	20	29.0 (27.3, 30.1)	0.588	26–34
MCHC, g/dL	10	35 (34, 36)	20	34 (33, 34)	0.01	31–36
RDW-CV, %	10	14.3 (13.0, 15.5)	20	13.6 (13.1, 15.0)	0.530	11.5–14.5
PLT, ×10^9^/L	10	83 (65, 176)	20	225 (212, 270)	0.006	* 150–400
MPV ×10^15^/L	10	10.7 (10.2, 11.2)	20	9.4 (9.2, 9.9)	0.003	7.0–12.0

The non-parametric Mann–Whitney U test was used to compare the hematological pediatric cancer group against healthy controls. WBC, white blood cells; HTC, hematocrit; RBC, red blood cells; HGB, hemoglobin; MCV, mean corpuscular volume; MCH, mean hemoglobin content; MCHC, mean hemoglobin concentration per red blood cell; RDW-CV, red blood cell distribution width, coefficient of variation; PLT, platelets; MPV, mean platelet volume; n/a—not applicable. * Ranges are defined for pediatric patients, from infants through adolescents.

**Table 2:** Identification of blood plasma metabolites in pediatric patients with ALL and healthy children using ^1^H NMR chemical shift data (ppm).

Plasma Analyte	ALL (*n* = 14) Median (ppm) (1st, 3rd Quartiles)	Controls (*n* = 20) Median (ppm) (1st, 3rd Quartiles)	*p* Level
Acetoacetate	1.38 (1.37, 1.40)	1.91 (1.91, 1.92)	0.125
Acetone	1.55 (1.54, 1.55)	2.24 (2.24, 2.24)	0.095
Alanine	1.01 (1.00, 1.02)	1.43 (1.42, 1.45)	0.193
Arginine	1.11 (1.11, 1.11)	1.58 (1.58, 1.58)	0.175
Citrate	1.80 (1.79, 1.81)	2.52 (2.52, 2.52)	0.071
Citrulline	2.21 (2.19, 2.23)	3.14 (3.14, 3.17)	0.038
Creatinine	2.12 (2.09, 2.12)	3.02 (3.02, 3.03)	0.044
Formate	5.84 (5.84, 5.84)	8.33 (8.33, 8.33)	<0.001
Glutamine	1.70 (1.69, 1.70)	2.44 (2.44, 2.44)	0.087
Glycerophosphocholine	2.27 (2.27, 2.27)	3.23 (3.23, 3.24)	0.035
Glycine	2.49 (2.49, 2.49)	3.55 (3.55, 3.55)	0.025
Guanosine monophosphate	5.69 (5.69, 5.70)	8.13 (8.13, 8.14)	<0.001
Histamine	5.35 (5.35, 5.38)	7.66 (7.65, 7.68)	<0.001
Isoleucine	0.64 (0.63, 0.66)	0.91 (0.91, 0.92)	0.294
Lactate	2.91 (2.90, 2.92)	4.17 (4.16, 4.17)	0.013
Leucine	0.64 (0.64, 0.67)	0.92 (0.92, 0.94)	0.288
Lysine	1.20 (1.19, 1.20)	1.79 (1.79, 1.79)	0.147
*Myo*-inositol	2.84 (2.83, 2.93)	4.06 (4.05, 4.06)	0.014
Phenylalanine	5.18 (5.18, 5.21)	7.46 (7.46, 7.46)	<0.001
Phosphocholine	2.25 (2.25, 2.27)	3.22 (3.22, 3.22)	0.036
*Scyllo*-inositol	2.35 (2.35, 2.35)	3.37 (3.37, 3.37)	0.031
Uridine diphosphate-glucose	3.97 (3.97, 3.97)	5.67 (5.67, 5.67)	0.002
Valine	0.73 (0.73, 0.75)	1.05 (1.04, 1.05)	0.265
α-H1 glucose	3.63 (3.61, 3.63)	5.17 (5.17, 5.18)	0.004
β-H2 glucose	2.30 (2.30, 3.31)	3.28 (3.28, 3.28)	0.033
β-hydroxybutyrate	0.84 (0.84, 0.85)	1.21 (1.21, 1.21)	0.233

Chemical shifts are reported in ppm. Median values are presented for comparison between cerebrospinal fluid and plasma samples. The non-parametric Mann–Whitney U test was used for statistical analysis between groups.

**Table 3:** Identification of cerebrospinal fluid and blood plasma metabolites in pediatric patients with ALL using ^1^H NMR chemical shift data (ppm).

Variable	CSF Median (ppm)	Plasma Median (ppm)	Wilcoxon Rank Sum	*p* Level
Acetone	2.25	2.22	48	0.060
Alanine	1.35	1.44	0	<0.001
Arginine	1.58	1.58	81.5	0.902
Citrate	2.57	2.57	37	0.031
Citrulline	3.17	3.16	70.5	0.382
Creatinine	3.04	3.03	60	0.177
Formate	8.34	8.34	35	1.000
Glutamine	2.43	2.43	88	0.822
Glycine	3.55	3.55	0	0.084
Lactate	4.02	4.15	19	0.002
Leucine	1.01	0.92	0	<0.001
Lysine	1.71	1.71	72	0.708
*Myo*-inositol	4.07	4.05	2.5	<0.001
*Scyllo*-inositol	3.36	3.36	0	0.157
Phenylalanine	7.2	7.4	0	<0.001
Valine	1.02	1.05	0	<0.001

Chemical shifts are given in ppm. Median values are presented to compare cerebrospinal fluid and plasma samples. The Wilcoxon signed-rank test was used for statistical analysis between groups.

**Table 4:** Chemical shifts of selected metabolites from the Human Metabolome Database, used for metabolite identification in cerebrospinal fluid and blood plasma.

Metabolites in Blood Plasma and CSF	Chemical Shift (ppm)	Metabolites in Blood Plasma	Chemical Shift (ppm)
Acetone	2.223	αH1-glucose	5.190
Alanine	1.455	βH2-glucose	3.280
Arginine	1.569	Glycerophosphocholine (GPC)	3.234
Citrate	2.537	Guanosine monophosphate (GMP)	8.220
Citrulline	3.161	Phosphocholine (PC)	3.226
Creatinine	3.041	Uridine diphosphate-glucose (UDP-gluc)	5.619
Formate	8.450		
Glucose	5.123		
Glutamine	2.428	Metabolites in CSF	Chemical shift (ppm)
Glycine	3.556	Acetate	1.910
Isoleucine	0.992	GABA	1.841
Lactate	4.107	Histidine	7.722
Leucine	0.998	2-Hydroxybutyrate	0.893
Lysine	1.721	3-Hydroxyisovalerate	1.193
*Myo*-inositol	4.070	Tyrosine	6.872
Phenylalanine	7.410		
*Scyllo*-inositol	3.360		
Valine	1.028		

Human Metabolome Database (http://www.hmdb.ca, accessed on 24 August 2023).

**Table 5:** Comparison of unique and common metabolites in the plasma and CSF of ALL patients with HMDB references.

Metabolite	Plasma	CSF	Relative Presentation (Chemical Shift)	HMDB Ref.
*Acetoacetate*	Yes	No	Unique to plasma	HMDB0000060
*Acetone*	Yes	Yes	Identical values in both samples	HMDB0001659
*Alanine*	Yes	Yes	Higher in CSF (1.35) than plasma (1.44) *	HMDB0000161
*Arginine*	Yes	Yes	Identical values in both samples	HMDB0000517
*Guanosine monophosphate*	Yes	No	Unique to plasma	HMDB0001397
*Leucine*	Yes	Yes	Lower in CSF (1.01) than plasma (0.92) *	HMDB0000687
*Myo-inositol*	Yes	Yes	Lower in CSF (4.07) than plasma (4.05) *	HMDB0000211
*Phenylalanine*	Yes	Yes	Higher in CSF (7.2) than plasma (7.4) *	HMDB0000159
*Phosphocholine*	Yes	No	Unique to plasma	HMDB0001565
*Valine*	Yes	Yes	Higher in CSF (1.02) than plasma (1.05) *	HMDB0000883

* Statistically significant differences (*p* < 0.001) suggest alterations in the microenvironment or the blood–brain barrier.

**Table 6:** Results of linear regression analysis assessing the correlation between age and metabolite signal intensities in the study cohort.

Metabolite	R^2^ Value	Regression Equation	Effect of Age
Formate	0.73	Formate concentration = 0.05 Age + 7.6	Strong, positive. Age is a key factor.
Arginine	0.15	Arginine concentration = −0.02 Age + 1.8	Weak, negative. Other factors are more significant.
Lactate	0.02	Lactate concentration = 0.01 Age + 1.2	No significant relationship. Concentration is not age-dependent.
Glucose	0.65	Glucose concentration = 0.08 Age + 5.0	Strong, positive. Age is a key factor influencing glucose concentration.
Valine	0.20	Valine concentration = 0.03 Age + 1.1	Weak, positive. Age has a moderate effect on valine concentration.
Glutamine	0.05	Glutamine concentration = −0.005 Age + 2.5	No significant relationship. The concentration is not age-dependent.

Linear regression analysis performed on simulated data allows for the assessment of the effect of age on the relative signal intensities of individual metabolites.

**Table 7:** Potential associations between experimental data and leukemia cell metabolic pathways.

Metabolite	Chemical Shift	Potential Metabolic Implications
Formate	Increased	Linked to Warburg effect and purine synthesis
Citrate	Increased	Disturbances in the Krebs cycle
Acetone	Decreased	Potential alterations in energy metabolism
β-hydroxybutyrate	Decreased	Potential alterations in energy metabolism
Glutamine	Decreased	High consumption for amino acid metabolism
Phenylalanine	Decreased	Disturbances in amino acid metabolism
Lysine	Decreased	Disturbances in amino acid metabolism
Myo-inositol	Decreased	Disturbances in carbohydrate metabolism
Scyllo-inositol	Decreased	Disturbances in carbohydrate metabolism
GPC	Increased	Altered lipid metabolism and membrane turnover

GPC, glycerophosphocholine.

## Data Availability

The authors would like to thank the Spectroscopy Laboratory of the Polish Academy of Sciences in Wrocław for providing access to the Bruker NMR AVANCE III™ 600 MHz spectrometer equipped with an Ascend™ magnet.

## Supplementary Materials

The following are available online at https://www.mdpi.com/article/10.3390/metabo16030160/s1.
